# Appetite Perception and Cerebral Blood Flow in Aging Adults Following a Single Bout of Exercise

**DOI:** 10.3390/nu18071072

**Published:** 2026-03-27

**Authors:** Steven K. Malin, Daniel J. Battillo, David H. Zald, Joslyn Ramirez

**Affiliations:** 1 Department of Kinesiology and Health, Rutgers University, New Brunswick, NJ 08901, USA; daniel.battillo@rutgers.edu (D.J.B.); jar595@scarletmail.rutgers.edu (J.R.); 2 Department of Medicine, Division of Endocrinology, Metabolism and Nutrition, Rutgers University, New Brunswick, NJ 08901, USA; 3 New Jersey Institute for Food, Nutrition and Health, Rutgers University, New Brunswick, NJ 08901, USA; 4 Institute of Translational Medicine and Science, Rutgers University, New Brunswick, NJ 08901, USA; 5 Center for Advanced Human Brain Imaging Research, Rutgers University, Piscataway, NJ 08854, USA; dz268@rbhs.rutgers.edu

**Keywords:** brain insulin resistance, type 2 diabetes, cerebrovascular disease, feeding behavior

## Abstract

Insulin acts in the brain to promote satiety. Aging individuals may have brain insulin resistance and altered appetite perceptions. However, it is unclear if exercise impacts cerebral reward centers and appetite perception in middle-aged to older individuals. **Purpose**: To assess whether a single exercise bout alters cerebral blood flow (CBF) in reward centers in relation to appetite perceptions. **Methods**: Fifteen sedentary adults (12F; ~56 ± 2y; ~31 ± 1 kg/m^2^) completed a control and acute exercise condition (70% maximal oxygen consumption) in a randomized, counterbalanced order in the evening. Following an overnight fast, CBF in the accumbens, thalamus, and amygdala (pCASL MRI) was evaluated before and after intranasal insulin spray (INI, 40 IU) administration. Plasma glucose and insulin as well as an appetite visual analog scale (VAS) were assessed at fasting, 30, and 90 min post-INI, as well as at 30 min intervals of a 120 min 75 g oral glucose tolerance test (OGTT). Total area under the curve (tAUC) was calculated. **Results**: Exercise tended to lower blood glucose (*p* = 0.072) and plasma insulin (*p* = 0.007) tAUC, compared with rest. Exercise also raised right thalamus (*p* = 0.029) and left amygdala CBF (*p* = 0.023). The rise in fasting CBF in these regions, and the accumbens, correlated with reduced insulin tAUC (r = −0.55 to −0.73, *p* < 0.050). Although there was no difference in hunger, satisfaction, fullness, or prospective food consumption after exercise, changes in INI-stimulated thalamus CBF related to fullness tAUC after exercise (r = −0.57, *p* = 0.044). **Conclusions**: A single exercise bout might increase fasting CBF in some brain regions associated with appetite perception through a potential insulin-related mechanism.

## 1. Introduction

Brain insulin sensitivity is important for not only memory, peripheral metabolism, and vascular function, but also food intake [1]. Intranasal insulin (INI) has been used to assess the mesocorticolimbic system, comprising of the accumbens, amygdala, hippocampus, striatum, thalamus, and prefrontal cortex, given its relation to food intake [2,3,4,5]. Interestingly, central insulin application reduces perceptions of hunger [6]. In contrast, reduced insulin action in the mesocorticolimbic system has been associated with desires for more palatable food in people with peripheral insulin resistance [2,3,4,5]. Aging people with obesity often develop hyperinsulinemia and insulin resistance, which can limit insulin transport to the brain via downregulation in the blood–brain barrier, and impair appetite. Thus, understanding how to combat appetite dysregulation in people with obesity is clinically relevant.

Caloric restriction and/or exercise are established lifestyle therapies that lower circulating insulin and improve peripheral insulin sensitivity [7]. However, limited studies exist that have assessed the impact of lifestyle therapies on brain insulin sensitivity. One trial showed that people with high brain insulin sensitivity prior to intervention lost more weight, including visceral fat, following diet plus exercise therapy in middle-aged adults compared with people who had low brain insulin sensitivity [8]. These findings are similar to others reporting that 12 weeks of caloric restriction of 10–15% increased brain insulin responsiveness compared with a waiting control group in older adults [6]. Interestingly, despite only modest weight loss of about 4% in this later study, gains in peripheral insulin sensitivity were related to strong inhibition of the nucleus accumbens during hedonic food assessment [6]. This suggests lifestyle therapy may favor appetite and weight regulation by an insulin-related brain mechanism. A consideration, nonetheless, of these aforementioned studies, is that they do not consider exercise per se, which has been shown to attenuate desires to eat during caloric restriction [9]. Although, it was recently reported that 8 weeks of aerobic exercise training reduced fasting hunger in parallel with INI-stimulated cerebral blood flow (CBF) in the putamen [10] among young, overweight adults with normoglycemia—people lost weight and enhanced fitness. These findings limit the understanding of exercise effects on the relationship between appetite perception and CBF. Moreover, the later study used 160 I.U. of INI. This dose is not used clinically, and if used, may increase risk for hypoglycemia in those who exercise [11,12]. Therefore, additional work investigating the effect of exercise prior to weight loss or fitness gains are needed to understand how appetite perception is impacted before and after a clinical dose of INI at 40 I.U. is required. Therefore, we tested the hypothesis that a single exercise bout performed the night before would favor appetite perception the next morning in middle-aged to older adults with excess body weight, and this improved appetite perception would correlate with changes in CBF among brain regions tied to reward centers.

## 2. Methods

### 2.1. Study Design and Participants

These were the same participants enrolled in prior studies examining the single bout effects of exercise on cognition and CBF responses to INI [13]. Herein, we focus on appetite and reference demographics as well as fasting glucose and insulin when appropriate to help with interpretations. Participants underwent a randomized, counterbalanced study using a computer-randomized sequence generated by a single investigator (DJB). People were recruited via social media, flyers, and/or electronic medical records from the local community between 2023 and 2025. Participants were excluded if physically active (>150 min/week of moderate intensity exercise), smoking, had an unstable weight > 2 kg over the last three months, taking insulin for glycemic control, had mild cognitive impairment (MCI)/dementia as assessed via the Montreal Cognitive Assessment (MoCA, <25 score), and had chronic disease (e.g., renal, hepatic, cardiovascular, etc.). A 12-lead electrocardiogram (EKG) was completed at rest and during maximal exercise to assess heart rhythms. A physician evaluated blood biochemistries and EKGs in conjunction with a physical to ensure eligibility. All women self-reported as post-menopausal except for one female who reported regular menses. In this later case, the participant completed the conditions about 30d apart in the follicular phase. This woman was not on oral contraceptives, nor did any woman who self-reported as post-menopausal take hormone replacement therapy. Study protocols conform to the Declaration of Helsinki and were approved by our Institutional Review Board (IRB#: 2022001842, approval date March 2023). People provided written and verbal consent. The study was registered on Clinicaltrials.gov (NCT# 05853913, registration date: May 2023).

### 2.2. Body Weight, Resting Metabolic Rate and Aerobic Fitness

Total body weight was recorded using a digital scale, and height was measured to the nearest 0.1 cm using a stadiometer to calculate body mass index. Resting metabolic rate (RMR) was determined with participants lying supine using a ventilated hood (COSMED Quark, Chicago, IL, USA) and data were averaged over the last 5 min for analysis to feed participants 24 h prior to the clinical visit. Aerobic fitness (VO_2_max) was assessed using indirect calorimetry on a treadmill as previously done by our laboratory [14].

### 2.3. Intervention Visits

Descriptions of our interventions have been previously reported [13]. In short, participants were asked to avoid alcohol, caffeine, and medication for 24 h prior to clinical testing. We used a randomized, counterbalanced design whereby people completed a rest and exercise condition on the night before assessments. The resting condition entailed 60 min of supervised seated rest completed at the same time as exercise to ensure minimal participant movement or activity. The exercise condition consisted of 5 min of resting indirect calorimetry to establish baseline measures followed by a 5 min warmup (45% VO_2_max) and 60 min of treadmill walking at 70% VO_2_max. Participants were provided standardized meals during the day of each intervention visit (~55% carbohydrate, 30% fat, and 15% protein) and instructed to eat their dinner at home after the intervention visit.

### 2.4. Clinical Testing and Cerebral Blood Flow

Participants arrived at the Center for Advanced Human Brain Imaging Research (CAHBIR) after a 10–12 h fast and approximately 15 h following the experimental conditions. When the participant arrived, they were placed in an upright chair for 5 min to rest in a quiet space. An IV line was then placed for assessment of fasting glucose, insulin, free fatty acids (FFA), lactate, and C-peptides. Appetite perception was then recorded using a visual analog scale (VAS, see below). Individuals were next taken to the MRI room. Using a 3 Tesla Siemens Prisma, they received a compressed sensing-MP2RAGE structural image (1 mm isotropic resolution) for spatial normalization and region of interest (ROI) definition [15]. CBF was measured using a pseudo-Continuous Arterial Spin Labeling (pCASL) sequence with a single post-labeling delay TE = 31.8 ms, TR = 4060 ms, labeling duration = 1509 ms, with 40 3 mm thick slices and 3.75 mm^2^ in plane resolution for whole brain coverage. After scanning, individuals were moved outside the MRI room and administered INI at a dose of 40 I.U. of human insulin (0.4 mL, Humulin, Eli Lilly & Co., Indianapolis, IN, USA) via VianaseTM electronic atomizers (Kurve Technology Inc., Lynnwood, WA, USA), as described before [13]. After 30 min, bloods were obtained again along with appetite perception. Scanning was repeated to determine post-INI CBF for brain insulin response assessment. Bloods and appetite were obtained after imaging (i.e., 90 min). Parameters and processing followed prior work for voxelwise calculation of CBF [16]. CBF was calculated with ASL-prep, which performs susceptibility distortion correction, motion correction, slice-timing correction, computes CBF using a one-compartment model and extracts CBF based on ROIs from co-registered atlas templates following spatial regularization partial volume correction (BASIL) [16]. Investigators were blinded to this analysis. Analysis of CBF focused on the left and right accumbens, thalamus, and amygdala defined by ROIs from the Human Connectome Project (HCP) subcortical atlas [17]. From prior work [13], we assessed here relations of appetite perception with putamen and caudate nucleus CBF given functions in reward processing and motivation. After CBF imaging, individuals underwent a 120 min 75 g oral glucose tolerance test (OGTT). Bloods and appetite were assessed every 30 min of the OGTT.

### 2.5. Appetite Assessment

Using a 100 mm VAS, perceived appetite was assessed as detailed previously [18]. Hunger, satisfaction, fullness, and prospective consumption (i.e., how much) were analyzed. Participants were asked to mark a single vertical line designating their subjective feelings on that occasion.

### 2.6. Biochemical Analyses

Plasma glucose and lactate were collected in vacutainers containing sodium fluoride. Plasma insulin, C-peptides, and FFA were obtained in EDTA vacutainers containing aprotinin and centrifuged at 4 °C for 10 min at 3000 RPM. Bloods were frozen at −80 °C until analysis. Plasma glucose and lactate were analyzed via oxidase methods (YSI Instruments 2500, Yellow Springs, OH, USA). Plasma insulin (ALPCO, Salem, NH, USA) and C-peptide (MilliporeSigma, Billerica, MA, USA) were analyzed via ELISA. FFAs were analyzed by colorimetric assays (FujiFilm Wako, Richmond, VA, USA).

### 2.7. Statistical Analysis

This was a secondary analysis to test effects of acute exercise on appetite perception following INI and OGTT responses from prior work [13]. No formal power analysis was conducted as described before [13], given such data were not available at the time of the study conception. However, we based enrollment in part on prior work we conducted that shows n = 12 adults undergoing the same exercise protocol had increased insulin sensitivity compared with resting conditions [14]. One participant who enrolled was unable to complete either rest or exercise conditions, leaving n = 15 for analysis and outcome reporting. SPSS (IBM, V. 28.0, Armonk, NY, USA) was used to analyze these data. A repeated measures analysis of variance (ANOVA) was used to assess differences between resting and exercise conditions. Additionally, total area under the curve (tAUC) was calculated via the trapezoidal model from 0 to 90 min to characterize INI effects, 90–210 min to depict OGTT effects, as well as 0–210 min to highlight overall protocol effects. Fasting and tAUC levels were then compared between conditions via a two-tailed paired *t*-test. To evaluate the physiological relevance among condition differences, Cohen’s d effect sizes for paired *t*-tests were calculated and interpreted as small d = 0.2, medium d = 0.5, or large d = 0.8. Partial eta squared (η^2^) was also calculated for repeated measures ANOVAs and interpreted as small η^2^ = 0.01, medium η^2^ = 0.06, or large η^2^ = 0.14. Spearman rank correlation was used to determine associations. Significance was accepted as *p* ≤ 0.05, and trends were accepted as *p* > 0.05 to *p* ≤ 0.10. Data are presented as mean ± SD.

## 3. Results

### 3.1. Participant and Exercise Characteristics

The participants’ (n = 15 (12F)) detailed characteristics were presented previously [13], and some data are shared here for ease of interpretation. Participants were middle-aged to older adults (~55y) with obesity (BMI = ~31 kg/m^2^) who had low aerobic fitness (VO_2_max = ~2.0 L/min). Individuals performed a single exercise bout at moderate to high intensity (HR = 157.8 ± 3.4 bpm, ~69% VO_2_max) and expended nearly 412 kcals.

### 3.2. OGTT

Compared with rest, exercise had no effect on fasting glucose (~98 vs. ~100 mg/dL) and insulin (~11 vs. ~11 µU/mL), or after INI as previously reported [13]. However, glucose during the OGTT and entire protocol tended to be lower after exercise compared with rest (both, *p* = 0.072, Table 1). These findings are independent of changes in fasting lactate (Rest: 1.2 ± 0.1 vs. Exercise: 1.1 ± 0.1 mmol/L, *p* = 0.282, d = 0.19) or fasting FFAs (Rest: 0.45 ± 0.07 vs. Exercise: 0.46 ± 0.04 mEQ/L, *p* = 0.868, d = 0.05). Similarly, there was no difference in lactate or FFA after INI, the OGTT, or the protocol overall (Table 1). In addition, insulin was significantly lower after exercise compared with rest during the OGTT (*p* = 0.004) and the entire protocol (*p* = 0.007). This reduction in insulin corresponds with a trend toward lower circulating C-peptides tAUC during the OGTT (*p* = 0.066, Table 1) independent of fasting C-peptides (Rest: 1.3 ± 0.7 vs. Exercise: 1.3 ± 0.7 ng/mL, *p* = 0.943, d = 0.01).

### 3.3. Appetite Perceptions

There was no statistical difference between rest and exercise in fasting hunger (39.8 ± 28.8 vs. 42.7 ± 34.6 mm, *p* = 0.617, d = 0.13), satisfaction (24.0 ± 18.3 vs. 24.9 ± 22.5 mm, *p* = 0.778, d = 0.07), fullness (22.3 ± 21.6 vs. 18.8 ± 23.8 mm, *p* = 0.438, d = 0.20), and prospective food consumption (50.7 ± 23.7 vs. 55.5 ± 27.2 mm, *p* = 0.169, d = 0.38, Table 2). There was also no effect of exercise on INI or OGTT hunger, fullness, and prospective food consumption responses either, when compared with rest (Table 2). Exercise did tend to lower satisfaction across the protocol compared with rest (*p* = 0.074, Table 2).

### 3.4. CBF and Correlations

**Figure 1:**
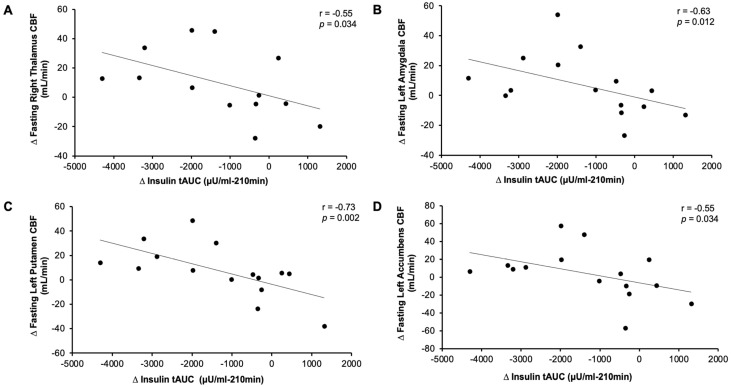
Association of insulin total area under the curve and cerebral blood flow. Note: Change exercise-rest (∆). Cerebral blood flow (CBF). Total area under the curve (tAUC).

**Figure 2:**
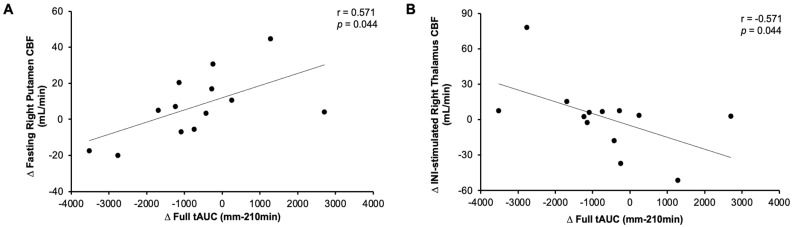
Association of fullness total area under the curve and cerebral blood flow. Note: Change exercise-rest (∆). Intranasal insulin (INI). Cerebral blood flow (CBF). Total area under the curve (tAUC).

Exercise raised CBF to the left amygdala (condition effect, *p* = 0.023), and this trended in the right amygdala (*p* = 0.062, Table 3). Exercise also raised CBF to the right thalamus (condition effect, *p* = 0.029), although this did not reach statistical significance in the left thalamus (*p* = 0.116, Table 3). These changes are likely driven by changes in fasting CBF, as no statistical effect of INI on CBF emerged in any region. There was no effect of exercise on the accumbens (Table 3) in the group as a whole. However, across individuals, the magnitude of change in fasting left and right accumbens CBF between rest and exercise inversely correlated with the degree of change in INI-stimulated CBF (i.e., CBF_30min post-INI_-CBF_0min_) (r = −0.91, *p* < 0.001 and r = −0.82, *p* < 0.001, respectively). Similarly, exercise-induced increases in fasting left and right thalamus CBF (r = −0.59, *p* = 0.02 and r = −0.68, *p* = 0.005) correlated with decreases in INI-CBF. Changes in fasting left and right amygdala CBF also inversely correlated with the change in INI-stimulated CBF (r = −0.52, *p* = 0.04 and r = −0.67, *p* = 0.007). Interestingly, increased fasting CBF in the right thalamus (r = −0.55, *p* = 0.034, Figure 1a) and amygdala (r = −0.63, *p* = 0.012, Figure 1b) as well as left putamen (r = −0.73, *p* = 0.002, Figure 1c) and accumbens (r = −0.55, *p* = 0.034, Figure 1d) correlated with a reduction in insulin tAUC_0–210min_. Reductions in insulin tAUC_0–210min_ also correlated with increased satisfaction tAUC_0–210min_ (r = −0.58, *p* = 0.027). Moreover, an increase in fullness tAUC for the entire protocol correlated with increased fasting right putamen CBF (r = 0.57, *p* = 0.044, Figure 2a) and decreased insulin-stimulated right thalamus CBF (r = −0.57, *p* = 0.044, Figure 2b).

## 4. Discussion

Previously, we reported that a single bout of exercise impacts CBF in brain regions associated with cognition [13]. Herein, we present new findings related to CBF in brain regions linked to appetite. The main finding from the present study is that a single exercise bout the night before had no effect on hunger, fullness, or prospective food consumption perception before or after INI, nor were there changes in response to glucose ingestion among aging adults. However, despite no effect of exercise on fasting, INI- or OGTT-related satisfaction, we did observe a trend toward slightly lower satisfaction across the protocol. While this may suggest less satiety during the protocol after exercise compared with rest, the overall subjective appetite response appears unchanged. Interestingly, exercise lowered circulating insulin during the protocol. This might be relevant, as insulin levels have been reported to relate to appetite perception [19,20,21]. Typically, insulin levels rise in the post-prandial state and contributes to reduced appetite. Lower insulin levels then might be expected to raise appetite responses. However, appetite perceptions were largely unchanged in our study after exercise, despite these lower insulin concentrations, potentially reflecting better whole-body insulin sensitivity. Prior work shows mixed results on the effect of INI and appetite. Indeed, administration of one dose of INI at 160 I.U. was reported to increase hypothalamus and orbitofrontal cortex activity among healthy lean women [3]. Similarly, INI lowered appetite, food intake, and palatability in healthy women during the post-prandial state only [22], which is consistent with other findings in women with obesity [23]. Interestingly, it has also been reported that women do not respond to INI at 160 I.U. with regard to decreased food intake acutely, and only men decreased body weight over time in response to insulin treatment [24]. Body weight status is an important factor when considering the disparate results across these studies, as generally those with excess weight do not respond to INI for lowering food intake [25,26]. Thus, our work is similar to some of this prior work reporting null appetite perception results [24]. Additional work in this area is warranted since studies have suggested that INI may favor appetite during the post-prandial state [22], and ambient insulin levels are known to influence appetite [19,20]. Our findings support this to some extent as ambient insulin correlated with CBF in regions related to appetite, and these regions were associated with fullness after exercise in middle-aged to older adults. While only a single exercise bout, these findings may have clinical relevance for understanding how exercise impacts appetite.

Various factors might help explain the impact of exercise on appetite perception in these participants. It is noteworthy that individuals were fed standardized isocaloric meals on the day of rest and exercise conditions. Subsequently, individuals who exercised expended on average ~412 kcal, which means they were in an energy deficit when measures were made. Often, it is described that energy deficits raise hunger and make it difficult to lose weight over time. However, individuals undergoing exercise did not raise their perception of hunger (i.e., higher VAS scores). These findings are similar to prior work by our group showing that exercise intensity had no effect on raising appetite [18]. Moreover, the present findings align with work from our lab showing that a short-term exercise training favored appetite perception while on a low-calorie diet in part through blunting acylated ghrelin responses to an OGTT [9]. While this prior work suggests ghrelin may play some role, FFA levels were elevated, and lactate as well as insulin concentrations were lower—in line with increased insulin sensitivity [27,28]. Prior work has suggested FFAs play a role on neural input to feeding [8,29,30]. Some studies report that INI reduces lipolysis [31] and plasma FFAs [32,33]. Thus, if FFA were lower throughout the protocol, this could impact appetite perception. However, FFAs were unaltered after INI or in response to glucose ingestion in the present work, suggesting there was no effect of exercise on FFAs to modulate appetite. Further, lactate has gained attention as an important substrate that potentially modifies appetite post-exercise [34]. However, we observed no difference in fasting, post-INI, or OGTT lactate responses throughout the protocol. Thus, the lack of difference in circulating lactate throughout the protocol following exercise compared with rest suggests that overall lactate is not likely to play a major factor in the present work.

Insulin acts on the brain to modulate hunger. Herein, we observed a subtle rise in fasting CBF to the amygdala and thalamus. Furthermore, the change in the fasting right amygdala and thalamus as well as left putamen and accumbens correlated with lower insulin tAUC throughout the protocol. This might be of physiologic relevance since lower insulin levels correlated with increased satisfaction, suggesting insulin related to exercise-mediated satiety. The reduction in circulating insulin in response to exercise is likely driven by elevations in peripheral insulin sensitivity and/or a combination of reduced pancreatic insulin secretion. The tendency of C-peptide to be lower during the OGTT with medium effect sizes suggests that insulin secretion to some extent may be lowered in response to gains in insulin sensitivity. This is relevant, as prior work suggests altered insulin secretion relates to CBF [35]. Herein, the increase in fasting CBF among our participants may be important too, as it correlates with lower INI-stimulated CBF after exercise, as previously reported [36,37]. This is also consistent with work showing decreased CBF after INI at 160 I.U. in regions involved in gustation and reward in young males who are overweight [38]. In our study, the relevance of these CBF changes could be important, since reductions in ambient insulin levels correlated with increases in fasting right amygdala and thalamus as well as left putamen and accumbens CBF. Why some individuals with reductions in circulating insulin had increases in fasting CBF compared with those who maintained or increased insulin is beyond the scope of this work. Yet individuals with obesity have been proposed to have brain insulin resistance, as evident by attenuated reward responses to insulin, thereby creating a potential feed-forward drive to continue consuming food [39]. Moreover, males who are overweight/obese have been characterized by higher CBF 30 min following administration of 160 I.U. INI in the prefrontal cortex versus lean people who showed reductions in INI-CBF as well as less desire for sweet foods [40]. Herein, we show that, as exercise raises fasting CBF, there is an inverse correlation with INI-stimulated CBF. This mirrors literature whereby lean people have higher brain glucose metabolism during the fasting state than people with obesity. In contrast, insulin stimulation during a clamp promotes brain glucose metabolism in people with obesity, suggesting that brain glucose metabolism is near maximum in lean people during fasting states [37]. Taken together, we show for the first time that exercise may favor increased fullness after exercise in relation with increased right fasting putamen CBF as well as decreased insulin-stimulated right thalamus CBF. Given these are regions tied to reward/motivation, our work suggests that a single exercise bout conducted the night before may have effects on the brain to influence feeding behavior in the morning.

This study has limitations that merit acknowledgement. This is a modest sample size, and associations do not equal causation. We also did not correct for multiple comparisons and accept an alpha of 0.05 as being statistically significant. No formal power analysis was performed given the nature of the work, and the outcomes should be interpreted with caution. Thus, larger studies are needed to confirm these findings and discern long-term training effects. Further, most participants were women, and women are generally considered to have higher brain insulin sensitivity than men. While this may limit gains seen with exercise, it is noteworthy that exercise favored increases in fasting CBF in some, but not all regions measured here and in our prior work [13]. This suggests the female participants measured in our work were at least somewhat responsive. Nonetheless, our modest sample size does not allow definite insights to potential sex-specific exercise effects [24,26,41] and further work is warranted. We also did not measure the effect of INI on ad libitum food intake given our design, nor did we identify the impact of exercise on appetite-related hormones (e.g., acylated ghrelin or GLP-1). With regard to the later hormones, prior exercise work we conducted shows no effect of exercise training for 2 weeks on either hormone during a 75 g OGTT when measured approximately 24 h after the last bout [18]. In contrast, when people consumed a low-calorie diet inducing about a 25% energy deficit, we noted that exercise attenuated rises in acylated ghrelin [8]. This suggests the degree of energy deficit may influence appetite-regulating hormones. Given that exercise in the present study was performed approximately 15 h before assessments of appetite and insulin and the energy deficit was modest, it would seem unlikely that these hormones played significant roles. However, we cannot discount the possibility that a combination of hormones, along with insulin versus insulin alone, contributed to changes in appetite perception during the OGTT. Further work assessing how exercise interacts with degrees of energy deficit would be helpful for understanding mechanisms related to the gut–brain axis for appetite control. Nonetheless, a strength of the work is that all people served as their own control, thereby allowing understanding of how exercise impacts appetite perception before and after INI as well as during an OGTT post-INI.

In conclusion, a single exercise bout conducted the evening before had no overall effect on appetite perception before or after INI, nor were there changes in response to glucose ingestion. We observed a subtle rise in fasting CBF to the amygdala and thalamus, and the change in the fasting right amygdala and thalamus as well as left putamen and accumbens correlated with lower insulin tAUC. These findings suggest that changes in peripheral insulin levels may influence CBF in regions related to appetite, such as satisfaction. This is supported to some extent, as increased fullness after exercise correlated with increased right putamen CBF as well as insulin-stimulated right thalamus CBF. While recent work suggests 40 I.U. of INI did not impact weight or appetite among aging adults, with and without type 2 diabetes [42], additional work exploring how exercise plus INI impact food intake and body weight overtime warrants attention, given the potential utility for impacting brain health.

## Figures and Tables

**Table 1:** Glucose, insulin, free fatty acids, lactate, and C-peptides responses to intranasal insulin and oral glucose ingestion.

	Rest	Exercise	*p*-Value	Cohen d
*Glucose* (mg/dL)				
tAUC_INI90-OGTT210min_	19,179.5 ± 918.7	17,943.0 ± 725.5	0.072	0.50
tAUC_0-OGTT210min_	27,897.5 ± 1093.2	26,615.3 ± 909.2	0.072	0.50
*Insulin* (µU/mL)				
tAUC_INI90-OGTT210min_	10,960.6 ± 1018.6	9622.6 ± 991.0	0.004	0.87
tAUC_0-OGTT210min_	11,794.2 ± 1104.0	10,488.2 ± 1075.2	0.007	0.81
*FFA* (mEQ/L)				
tAUC_0-INI90min_	47.1 ± 5.0	48.3 ± 3.5	0.800	0.07
tAUC_INI90-OGTT210min_	33.9 ± 3.8	35.2 ± 3.4	0.643	0.13
tAUC_0-OGTT210min_	81.0 ± 8.3	83.5 ± 6.3	0.729	0.10
*Lactate* (mmol/L)				
tAUC_0-INI90min_	81.2 ± 7.3	83.0 ± 8.7	0.765	0.07
tAUC_INI90-OGTT210min_	137.5 ± 12.4	133.3 ± 15.6	0.860	0.04
tAUC_0-OGTT210min_	219.5 ± 19.9	221.9 ± 24.5	0.850	0.04
*C-peptides* (ng/mL)				
tAUC_0-INI90min_	109.3 ± 52.9	109.7 ± 56.4	0.945	0.01
tAUC_INI90-OGTT210min_	669.1 ± 179.1	629.7 ± 185.5	0.066	0.51
tAUC_0-OGTT210min_	778.4 ± 221.3	739.4 ± 224.7	0.112	0.43

Note: Data are mean ± SD. Data were analyzed by *t*-tests. Fasting glucose and insulin data were previously presented, thus only oral glucose tolerance test (OGTT) and protocol data are reported. Free fatty acids (FFA). Total area under the curve (tAUC). Intranasal insulin (INI).

**Table 2:** Appetite responses to intranasal insulin and oral glucose ingestion.

	Rest	Exercise	*p*-Value	Cohen d
*Hungry* (mm)				
tAUC_0-INI90min_	4628.0 ± 2294.0	4746.5 ± 2527.2	0.693	0.10
tAUC_INI90-OGTT210min_	6983.5 ± 2608.6	7379.8 ± 2741.8	0.245	0.32
tAUC_0-OGTT210min_	12,033.2 ± 4800.3	12,703.2 ± 5083.0	0.304	0.07
*Satisfied* (mm)				
tAUC_0-INI90min_	2062.0 ± 1724.3	1848.9 ± 1576.5	0.268	0.29
tAUC_INI90-OGTT210min_	2751.0 ± 1715.8	2191.9 ± 1518.6	0.327	0.26
tAUC_0-OGTT210min_	4958.5 ± 3494.6	4110.9 ± 2972.1	0.074	0.51
*Full* (mm)				
tAUC_0-INI90min_	1702.0 ± 1236.2	1548.3 ± 1546.7	0.504	0.17
tAUC_INI90-OGTT210min_	2299.0 ± 1519.2	2191.9 ± 1518.6	0.783	0.07
tAUC_0-OGTT210min_	4062.9 ± 2804.9	3478.4 ± 2692.5	0.144	0.43
*Much* (mm)				
tAUC_0-INI90min_	5280.0 ± 1913.7	5544.8 ± 2013.0	0.386	0.23
tAUC_INI90-OGTT210min_	7439.0 ± 2375.6	7865.4 ± 2391.6	0.231	0.32
tAUC_0-OGTT210min_	13,013.6 ± 4401.4	13,916.6 ± 4233.5	0.276	0.31

Note: Data are mean ± SD. Data were analyzed by *t*-tests. Intranasal insulin (INI). Oral glucose tolerance test (OGTT). Total area under the curve (tAUC).

**Table 3:** Cerebral blood flow before and after intranasal insulin with and without exercise.

	Rest		Exercise					
	0 min	INI 30 min	0 min	INI 30 min	C	T	CxT	η^2^
*Left* (mL/min)								
Amygdala	85.23 ± 40.25	79.59 ± 32.64	91.89 ± 31.76	94.26 ± 35.59	0.023	0.720	0.381	0.12
Accumbens	73.23 ± 31.06	72.41 ± 30.86	77.27 ± 26.61	78.03 ± 26.35	0.314	0.995	0.869	0.02
Thalamus	52.23 ± 7.16	49.48 ± 5.23	57.20 ± 8.23	57.34 ± 8.21	0.116	0.746	0.720	0.06
*Right* (mL/min)								
Amygdala	84.69 ± 37.68	81.01 ± 37.37	92.65 ± 38.53	91.57 ± 39.80	0.062	0.626	0.790	0.08
Accumbens	65.98 ± 29.55	65.48 ± 30.82	72.43 ± 22.93	72.77 ± 23.81	0.138	0.985	0.926	0.05
Thalamus	55.49 ± 7.70	52.85 ± 6.67	65.07 ± 8.43	60.31 ± 8.84	0.029	0.333	0.780	0.11

Note: Data are mean ± SD. Data were analyzed by ANOVA. Condition = C, rest vs. exercise. Time = T, 0 vs. 30 min comparison. Intranasal insulin (INI).

## Data Availability

These data have not been made publicly available. However, the corresponding author can provide further information on the data upon reasonable request.
